# Meningoencephalitis Associated with SARS-Coronavirus-2

**DOI:** 10.37825/2239-9747.1007

**Published:** 2020-10-01

**Authors:** G Iaconetta, P De Luca, A Scarpa, C Cassandro, E Cassandro

**Affiliations:** 1 Department of Medicine, Surgery and Dentistry, University of Salerno, Salerno, Italy; 2 Department of Surgical Sciences, University of Turin, Turin, Italy

**Keywords:** Meningitis, Encephalitis, COVID-19, Sars-Coronavirus-2, neurological complications

## Abstract

The aim of this work is to clarify the incidence of meningitis/encephalitis in SARS-CoV-2 patients. We conducted an initial search in PubMed using the Medical Subject Headings (MeSH) terms “meningitis,” and “encephalitis,”, and “COVID-19” to affirm the need for a review on the topic of the relationship between meningitis/encephalitis and SARS-CoV-2 infection. We included case series, case reports and review articles of COVID-19 patients with these neurological symptoms. Through PubMed database we identified 110 records. After removal of duplicates, we screened 70 record, and 43 were excluded because they focused on different SARS-CoV-2 neurological complications. For eligibility, we assessed 27 full-text articles which met inclusion criteria. Seven articles were excluded, and twenty studies were included in the narrative review, in which encephalitis and/or meningitis case reports/case series were reported.

Neurological manifestations of COVID-19 are not rare, especially meningoencephalitis; the hypoxic/metabolic changes produced by the inflammatory response against the virus cytokine storm can lead to encephalopathy, and the presence of comorbidities and other neurological diseases, such as Alzheimer’s disease, predispose to these metabolic changes. Further study are needed to investigate the biological mechanisms of neurological complications of COVID-19.

## I. INTRODUCTION

The Severe Acute Respiratory Syndrome-CoronaVirus-2 (SARS-CoV-2) was firstly and officially identified on January 2020 in Wuhan, China, from a patient with atypical pneumonia^1^; the COVID-19 global pandemic was declared two months later, affecting millions of people and engaged clinicians around the world in an unprecedented effort to limit the viral spread and treat affected patients^2^. As of August 18, 2020, the COVID-19 pandemic has resulted in more than 22 million confirmed cases worldwide and more than 780.000 deaths. SARS-CoV-2 primarily affects the respiratory tract; the most common clinical symptoms are fatigue, cough, shortness of breath, and mainly respiratory tract symptoms^3^.

Coronaviruses are respiratory viruses with neurotropic capacities, which allow them to avoid the immune response; viral RNA has been found in the cerebrospinal fluid, after SARS-CoV exposure, in patients with encephalitis, ischemic stroke and polyneuropathy^4–6^. Neurological SARS-CoV-2 virus-associated symptoms are not uncommon, and an increasing number of cases have presented with neurologic manifestations (such as headache, anosmia, taste disorders, Guillan-Barrè syndrome, and meningoencephalitis) and can be devastating complications^7,8^; the common neurological symptoms included olfactory (60.6%) and gustatory (61%) disorders, especially in mild cases^9^. In one of most representative case series from Wuhan, China, 36.4% of patients had neurological complications^10^.

The pathophysiological mechanisms have not been completely established, but the hypoxic/metabolic changes, produced by the inflammatory response against the virus cytokine storm, can lead to encephalopathy^11–12^. The SARS-CoV-2 infection involves the central nervous system due to the direct neuroinvasion and, mainly, the neurological sequelae due to the systemic innate-mediated hyper-inflammation. The possible routes of neuroinvasiveness of SARS-CoV-2 are well summarized by Khan and Gomes^13^; the hematogenous route, through which the virus infects endothelial cells of the blood-brain barrier and invades neuronal tissue, and the neuronal retrograde spread, via olfactory nerves from the nasal cavity through cribriform plate, the virus reaching the central nervous systems, are the most accepted theories^14^. Besides, a recent theory suggested that neuroinvasion via the enteric pathway can be possible, due to the enteric nervous system invasion or reaching the central nervous system via intestinal vagal afferents^15^.

Low levels of Angiotensin-converting enzyme 2 (ACE2), a functional receptor for the SARS-CoV-2, has been detected in the human brain^16^; moreover, a recent study demonstrated that the brain is the principal target organ for SARS-CoV in transgenic mice for human ACE2 receptor^17^.

Moreover, a potential role of the spike glycoprotein (S1)-S1 IgG antibody complex was hypotized to facilitate the entry of the virus into the host immune cells^18^.

The purpose of this brief review is to clarify the incidence of meningitis/encephalitis SARS-CoV-2 associated.

## II. METHODOLOGY

### 2.1 Search Strategy and Study Selection

We conducted an initial search in PubMed using the Medical Subject Headings (MeSH) terms “meningitis,” and “encephalitis,”, and “COVID-19” to affirm the need for a review on the topic of the relationship between meningitis/encephalitis and SARS-CoV-2 infection.

The research protocol was designed to define the objective and the methodology of the study; the protocol was neither registered nor published.

The search strategy was chosen in consultation with a senior librarian at the University of Salerno (Salerno, Italy). A comprehensive review of the English-language literature was performed by using the following databases: the National Library of Medicine’s version of PubMed (1988 to June 2020). The last search was performed on August 16, 2020.

We included case series, case reports and review articles of COVID-19 patients affected by neurological symptoms. After exclusion of duplicates, all articles were evaluated through title and abstract screening by two independent reviewers (Pietro De Luca and Giorgio Iaconetta). The same two reviewers performed an accurate reading of all full-text articles assessed for eligibility, and performed a collection of data to minimize the risk of bias.

Inconsistencies regarding inclusion of a given study were adjudicated by a third reviewer (Ettore Cassandro).

## III. RESULTS

Through PubMed database we identified 110 records. After removal of duplicates, we screened 70 record, and 43 were excluded because they focused on different SARS-CoV-2 neurological complications. For eligibility, we assessed 27 full-text articles which met inclusion criteria. Seven articles were excluded for the following reasons: a) Articles in Polish (n= 1), b) Article in Chinese (n= 6). Twenty studies were included in the narrative review, in which encephalitis and/or meningitis case reports/case series were reported.

## IV. DISCUSSION

The SARS-CoV-2 infection is not only a respiratory disease; neurological involvement is not rare. This has been clear from the first analysis of 214 cases of COVID-19 in Wuhan (China), where 36.4% (78) patients presented with neurological complications^19^.

Several cases of meningoencephalitis have been reported since February 2020, as shown in Tab. 1.

The first case of encephalitis with SARS-CoV-2 isolated in cerebrospinal fluid was reported by Moriguchi and colleagues on February 2020; this was the case of a 24-year-old man who was found lying on the floor with consciousness disturbance (GCS 6) nine days after he felt headache and generalised fatigue. This was the first case report in which the Authors highlighted the possibility of a neuroinvasive potential of COVID-19^20^.

Bernard-Valnet et al. reported the clinical evolution of two patients, a 64-year-old woman and a 67 year-old man, who developed meningoencephalitis after several days of mild respiratory symptoms; both patients showed a significant improvement 24 hours after admission^21^.

A combination of altered consciousness, focal neurological defects, fever, and cerebrospinal fluid abnormalities in a 69-year-old man one week after the onset of COVID-19 was described by El Otmani et al^22^. They reported that the detection of SARS-CoV-2 in bronchoalveolar lavage as the time of lumbar puncture was in favour of a direct infection mechanism.

In addition, Pilotto et al^23^ described the case of a 64-year-old man with a mild respiratory impairment who developed a kinetic mutism due to an encephalitis that highly improved after an high-dose steroid treatment. A case of SARS-CoV-2 infection who presented with isolated encephalitis was reported by Ye et al^24^.

Moreover, the first pediatric case was described by McAbee et al^25^, after two days of generalized weakness, without respiratory symptoms, he presented with epileptic seizures requiring four anticonvulsant medications.

Also atypical presentations of neurological involvement are reported. A COVID-19 associated encephalitis mimicking glial tumor was described by Efe et al.; the Authors suggested that severe neurologic manifestations of COVID-19 could mimic this brain tumors^26^. A mild encephalitis, in a 75-year-old man with a history of mild Alzheimer’s disease, was observed with a reversible splenial lesion^27^. A unique case of neurological symptoms developed before clinical and pulmonary manifestations, has been described by Abdi et al^28^.

Kremer et al retrospectively reviewed the characteristics of 64 confirmed COVID-19 patients with neurological complications, who underwent a brain MRI; encephalitis was one of the most common neuroimaging finding (17%); moreover, patients with encephalitis were younger. In this work, the Authors were incline to accept the autoimmune/inflammatory theory, due to the absence of the virus in cerebrospinal fluid and the presence of signs of inflammation in both neuroimaging and cerebrospinal fluid^29^. We found 4 systematic review aiming to investigate the evidence of neurological impairment in COVID-19. Ghannam et al. reported that 19 patients (23%) had encephalitis or encephalopathy^30^; on the contrary, Tsai et al. reviewed only 4 case reports about meningoencephalitis in COVID-19 patients^31^.

Romoli et al. reported a similar rate, and they suggested to perform, in case of suspected neuroinvasion, PCR testing for SARS-CoV-2 in cerebrospinal fluid and anti-SARS-CoV-2 IgM-IgG testing in serum, and cerebrospinal fluid to check for intrathecal humoral immune reaction^32^. Moreover, recently the presence of SARS-CoV-2 RNA in the cerebrospinal fluid has been detected by genome sequencing in a patient with clinically proved meningoencephalitis in Japan^33^.

The direct invasion, via hematogenous pathways, is considered by Wang et al. to be responsible for the rapid progression of neurological symptoms in patients with encephalitis [**6**]. In Agarwal et al. opinion’s, the encephalitis could be due to the direct effect of the virus on the brain, or secondary to immune-mediated changes as seen with H1N1^34^.

It is actually unknown if the COVID-19 infection could *per se* represent an independent risk factor for the developing of neurological complications^35^. Pre-existing disorders of the central nervous system may play a role in exacerbating the disease, and may be an important risk factor for increased pulmonary complications. This question is supported by Arg et al^36^, who suggested that the presence of neurological disease predisposes to hypoxic/metabolic changes and to encephalopathy; in addition they found that encephalitis is common in older patients (>50-year-old), in patients critically ill, and most of these patients presented lung abnormalities and many of them were already on mechanical ventilation, or in the intensive care unit when encephalopathy developed. Besides, cortical and subcortical T2/FLAIR signal changes are common neuroimaging abnormalities.

Moreover, several Authors confirmed that, in some cases, the neurological symptoms can even precede the respiratory ones or can be the only symptoms in COVID-19 patients^37^.

An earlier recognition of a neurological impairment in patients with a mild or asymptomatic respiratory infection can be challenging, also to prevent the long-term neurological sequelae and to contain social and economic costs^38^

## V. CONCLUSION

Neurological manifestations of COVID-19 are not rare, especially meningoencephalitis, which could be a devastating SARS-CoV-2 associated Central Nervous System complications, especially in older and critical patients, in which can cause lifelong disability and death. The hypoxic/metabolic changes produced by the inflammatory response against the virus cytokine storm can lead to encephalopathy, and the presence of comorbidities and other neurological diseases (as Alzheimer’s disease) predispose to these metabolic changes. Also the autoimmune response can play a role in the pathogenesis of brain damage related to SARS-CoV-2. Further studies are needed to investigate the biological mechanisms of neurological complications of COVID-19.

## Figures and Tables

**Table 1:** Clinical characteristics of meningoencephalitis case-report

Author, Country	Number of meningoencephalitis observed	Clinical presentation	Outcome
**Efe et al, Germany**	1	A 35-year-old woman with headache and seizure. The MRI was suggestive of high-grade glioma. A left anterior temporal lobe was performed. histologic examination revealed encephalitis	No postoperative deficits, and her symptoms completely improved
**Abdi et al, Iran**	1	A 58-year-old woman presented with decreased level of consciousness and the inability to walk. Brain MRI indicated differ confluent white matter hyperintensity on FLAIR-weighted-MRI at the left side	After 10 days, status epileptics developed and the patient died one day later
**Bernard-Valnet et al, Switzerland**	2	A 64-year-old woman presented a tonico-clonic seizure; MRI was normal, but lumbar puncture was compatible with viral meningoencephalitis and SARS-CoV-2 was detected in her nasopharyngeal swab A 67-year-old woman, already diagnosed with SARS-CoV-2 infection for 17 days with mild respiratory symptoms, presented an intense wake-up headache, bilateral grasping, aggressiveness and left hemianopia and sensory hemineglect.	The patient markedly improved 96 h after admission with resolution of her symptoms Neurological symptoms resolved within 24 h, except for a mild headache. The patient was discharged 72 h after admission with no symptoms
**McAbee et al, United States**	1	A 11-year-old child presented with status epileptics; he had a two-day history of generalized weakness without respiratory symptoms. Electroencephalography revealed frontal intermittent delta activity.	Recovery without treatment was complete in six days
**Ye et al, China**	1	A Wuhan male, who previously had fever, shortness of breath and myalgia, presented with his consciousness progressed to confusion. Meningeal irritation signs and extensor plantar response were present.	The patient was discharged 17 days after admission
**El Otmani et al, Morocco**	1	69-yr-old man with a 7-day history of fever and cough, was admitted for confusion and severe headache; brain MRI was normal.	At discharged on day 10, mild neuropsychiatric features were still present with an alteration of executive functions
